# Estimating the economic burden of influenza on the older population in Malaysia

**DOI:** 10.1371/journal.pone.0294260

**Published:** 2023-11-16

**Authors:** Nur Syazana Mad Tahir, Aniza Ismail, Syed Mohamed Aljunid, Aznida Firzah Abdul Aziz, Amirah Azzeri, Ahmed Abdelmajed Alkhodary

**Affiliations:** 1 Department of Public Health Medicine, Faculty of Medicine, Universiti Kebangsaan Malaysia, Cheras, Kuala Lumpur, Malaysia; 2 Ministry of Health Malaysia, Federal Government Administrative Centre, Putrajaya, Malaysia; 3 International Centre for Casemix and Clinical Coding, Hospital Canselor Tuanku Muhriz, Universiti Kebangsaan Malaysia, Cheras, Kuala Lumpur, Malaysia; 4 Department of Public Health and Community Medicine, School of Medicine, International Medical University, Bukit Jalil, Kuala Lumpur, Malaysia; 5 Department of Family Medicine, Faculty of Medicine, Universiti Kebangsaan Malaysia, Cheras, Kuala Lumpur, Malaysia; 6 Public Health Unit, Department of Primary Health Care, Faculty of Medicine and Health Sciences, Universiti Sains Islam Malaysia, Nilai, Negeri Sembilan, Malaysia; 7 Ministry of Health—Gaza governorates, Gaza, Palestine; University of Cyberjaya, MALAYSIA

## Abstract

**Background:**

Influenza is a contagious respiratory illness that can cause life-threatening complications among high-risk groups. Estimating the economic burden of influenza is essential to guide policy‐making on influenza vaccination programmes, especially in resource‐limited settings. This study aimed to estimate the economic burden of influenza on older adults (those aged ≥60 years) in Malaysia from the provider’s perspective.

**Methods:**

The main data source in this study was the MY-DRG Casemix database of a teaching hospital in Malaysia. Cases with principal and secondary diagnoses coded in the International Classification of Diseases version 10 (ICD-10) as J09, J10.0, J10.1, J10.8, J11.0, J11.1, J11.8, J12.8, and J12.9, which represent influenza and its complications, were included in the study. The direct cost of influenza at all severity levels was calculated from the casemix data and guided by a clinical pathway developed by experts. The effect of the variations in costs and incidence rate of influenza for both the casemix and clinical pathway costing approaches was assessed with sensitivity analysis.

**Results:**

A total of 1,599 inpatient and 407 outpatient influenza cases were identified from the MY-DRG Casemix database. Most hospitalised cases were aged <18 years (90.6%), while 77 cases (4.8%) involved older people. Mild, moderate, and severe cases comprised 56.5%, 35.1%, and 8.4% of cases, respectively. The estimated average annual direct costs for managing mild, moderate, and severe influenza were RM2,435 (USD579), RM6,504 (USD1,549), and RM13,282 (USD3,163), respectively. The estimated total annual economic burden of influenza on older adults in Malaysia was RM3.28 billion (USD782 million), which was equivalent to 10.7% of the Ministry of Health Malaysia budget for 2020. The sensitivity analysis indicated that the influenza incidence rate and cost of managing severe influenza were the most important factors influencing the total economic burden.

**Conclusions:**

Overall, our results demonstrated that influenza imposes a substantial economic burden on the older Malaysian population. The high cost of influenza suggested that further efforts are required to implement a preventive programme, such as immunisation for older people, to reduce the disease and economic burdens.

## Introduction

Influenza viruses are a significant public health concern that can cause acute respiratory illness. In the Northern and Southern Hemispheres, influenza commonly occurs during the winter season, but occurs year-round in tropical countries that have no clear seasonal pattern, typically with several peaks during rainy seasons with irregular outbreaks [1]. The World Health Organisation (WHO) reported that seasonal influenza contributes to a year-round disease burden that causes an illness with various severity levels that can lead to hospitalisation and death [2]. For example, Canada recorded influenza-related hospitalisation of 23%, and 15% of inpatient deaths were related to influenza [3]. A recent report from the US estimated that the 2021–2022 influenza season recorded nine million illnesses, four million medical visits, 10,000 hospitalisations, and 5,000 deaths. The burden of influenza cases was comparable to that reported in 2011–2012 [4]. Although influenza might be harmless in the long-term, the accompanying complications might be related to life-threatening medical conditions, especially in high-risk populations, as illustrated by the 2017 Global Burden of Disease Study [5].

The estimated Malaysian influenza-related excess mortality rate was 37.9 and 111.9 per 100,000 in people aged 65–74 years and ≥75 years, respectively [6]. Due to their lower immune systems, older adults are significantly more susceptible to severe influenza and influenza complications, and the infection itself can exacerbate underlying conditions, increasing the risk of hospitalisation and death. Older people were more severely affected by influenza than younger people, where older people accounted for 10–25 times more hospitalisations than younger patients annually [7, 8]. The WHO recommended a 50–90% and 60% vaccine coverage rate for older adults and high-risk adults, respectively [9]. In Malaysia, influenza vaccines are widely available but are not funded under the National Immunisation Programme. Although influenza vaccination is an inexpensive and effective means of preventing illness and minimising productivity losses, the level of adoption in society has been too low to achieve even a tiny percentage of its potential value. The out-of-pocket price for influenza vaccine ranged from RM41-100 (USD10-24). However, older persons demonstrated low rates of vaccine uptake, which can be attributed to low awareness, lack of urgency and unwillingness to bear the cost of influenza vaccines [10].

The fundamental goal of cost of disease studies is to evaluate the total burden imposed by a disease on society as a whole. Estimating the economic burden is essential to guide policy‐making on influenza vaccination programmes, especially in resource‐limited settings. A current study from the US estimated that the annual total economic burden of influenza on the healthcare system was approximately USD11.2 billion, with USD3.2 billion estimated from direct medical costs alone [11]. However, data on the direct medical costs or studies on the cost analysis of influenza in Malaysia are limited. The cost analysis estimates the actual price to treat and manage the patient. In health economics, the cost analysis estimates the economic burden of a particular disease on society. Thus, it can aid the selection of the most effective treatment.

Therefore, the main objective of this study was to estimate the economic burden of influenza in older adults (age ≥ 60 years) from the provider’s perspective. The economic burden was estimated with the direct cost from MY-DRG Casemix data and clinical pathway costing analyses by considering the disease severity level. A recent Malaysian study detected influenza in 10.3% of the adult population hospitalized with community-acquired pneumonia, acute exacerbation of chronic obstructive pulmonary disease, or acute exacerbation of asthma [12]. Malaysian influenza rates are comparable with those previously described in East and Southeast Asia, where estimates ranged 5–14% of the study population [13, 14].

## Methods

### Cost estimates of influenza

In this study, the primary data source was the MY-DRG Casemix database of a teaching hospital in Malaysia. The Casemix System is a classification system that groups patients with similar clinical characteristics, which are homogenous in terms of the resources used and a more significant outcome [15]. The Diagnosis-Related Groups (DRGs) were the first grouping system developed under the casemix system based on the International Classification of Diseases, version 10 (ICD-10) and the International Classification of Diseases, version 9, Clinical Modification (ICD-9-CM) to measure hospital costs [15]. Cases with principal or secondary diagnoses coded in the ICD-10 as J09, J10.0, J10.1, J10.8, J11.0, J11.1, J11.8, J12.8, and J12.9, which represent influenza and its complications, were included in the study. A total of 1,599 inpatient cases and 407 outpatient cases were included in the study. The inpatient case data were distributed from 2010 to 2020, while the outpatient case data were from 2019 and 2020; this was the only existing outpatient data set at this hospital. Data were accessed on March 5, 2022 from the casemix database. This study was conducted after obtaining ethical clearance from the research committee of the Universiti Kebangsaan Malaysia (UKM) (UKM PPI/111/8/JEP-2021-603) and the Medical Research Committee, Faculty of Medicine, UKM (FF-2021-353). The study does not require informed consent because the data was extracted from MY-DRG database and analysed anonymously which did not involve interviews of patients.

Cost analysis was also carried out using a previously published clinical pathway for influenza based on the service care type and severity [16]. The treatment cost per patient was estimated with a combination of step-down and activity-based costing. Step-down costing comprises three cost centres: overhead, intermediate, and final. The information on the floor space area for each cost centre and the annual hospital expenditure cost, which includes building, utility, maintenance, and machine costs and staff salary, were acquired from a teaching hospital in Malaysia. Subsequently, the calculated cost was multiplied by the patient’s length of stay (LOS) in the medical ward to determine the inpatient cost per disease episode. For activity-based costing, the consumable, drug, procedure, and investigation costs were calculated from the listed activity in the clinical pathway for influenza [16]. Similarly, the estimation for outpatient cost per patient was based on the developed clinical pathways for outpatient mild and moderate influenza. The operational cost was calculated using step-down costing, while the estimation of the medical and investigation costs was calculated from the listed activity in the clinical pathways [15]. For outpatient moderate influenza, the calculated cost was multiplied by two visits [15]. The total cost for inpatient and outpatient per episode of care was calculated by summing the step-down and activity-based costings.

The annual costs for severe influenza with pneumonia and moderate cases were estimated by summing the direct inpatient and moderate outpatient costs as recommended by the experts. The experts in family medicine, public health, geriatric, respiratory and infectious disease were involved in the decision-making and recommendation processes [15]. Indirect costs such as lost productivity and absenteeism were not accounted for in this estimation. Our primary aim was to estimate the direct medical costs, which reflects the provider perspective as they relate more directly to the allocation of healthcare resources and budget planning from policymakers. The cost in US dollars (USD) was interpreted using the 2020 average exchange rate for the Malaysian ringgit.

### Total economic burden of influenza

We calculated the total economic burden of influenza for both the casemix and clinical pathway costing methods. For casemix costing, the cost for patients aged ≥60 years was selected to estimate the annual cost per older adult influenza patient in Malaysia. Each patient was assumed to have two outpatient visits in 1 year prior their admission to the hospital. This assumption was established according to the moderate influenza outpatient clinical pathway developed previously [16]. The published incidence rate for influenza hospitalisations in 2019 [12] was applied to the older population in Malaysia [17] to estimate the number of hospital admissions attributable to influenza. We calculated the weighted cost per episode for mild, moderate, and severe influenza by multiplying the annual cost per patient with the percentage of older patients according to the severity levels in casemix database. Subsequently, the weighted cost was multiplied by the estimated number of hospitalisations to calculate the total annual cost of influenza in the older population. These methods and assumptions were also applied to the clinical pathway costing to calculate the total economic burden of influenza. However, one visit was assumed for mild influenza following the clinical pathway for outpatients of this severity [16]. Finally, the total economic burden of influenza among the older adults in Malaysia was estimated using the average cost from both costing approaches by severity.

### Sensitivity analysis

**Fig 1:**
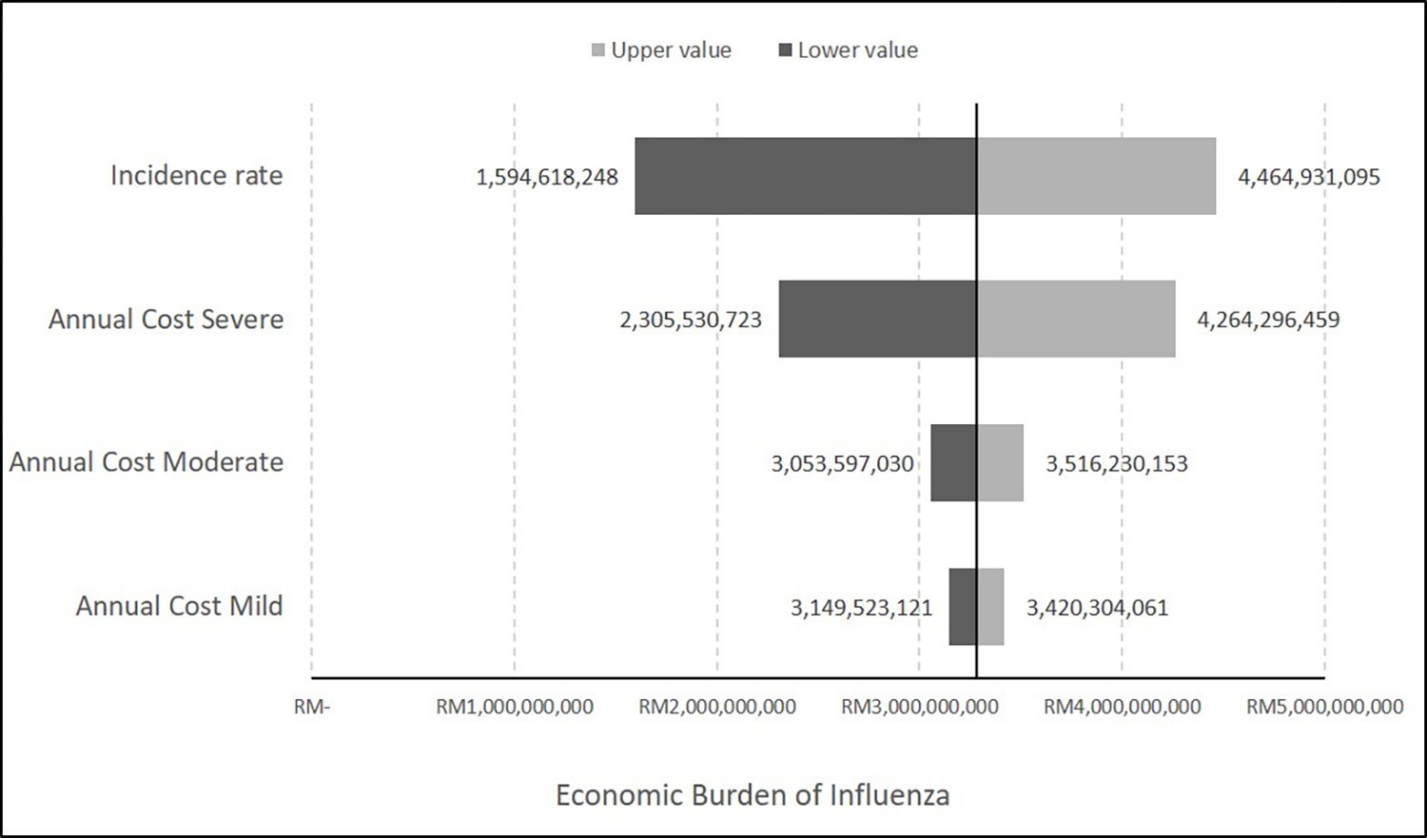
Tornado diagram result from one-way sensitivity analysis.

The influence of uncertainty on the economic burden of influenza was assessed with sensitivity analysis (Table 1). We conducted a one-way sensitivity analysis in which the costs of mild, moderate, and severe influenza varied between the casemix and clinical pathway costing methods. The effect of the different influenza incidence rates was examined using the range from previously published literature, i.e. 5–14% [13, 14]. The average annual costs from the casemix and clinical pathway costing methods for mild, moderate, and severe influenza were used as the baseline values. The value from a recent publication [12] was used as the baseline for the influenza incidence rate. The results are presented in tornado diagrams based on the total economic burden of influenza (Fig 1). A tornado diagram is a specific bar chart that presents the graphical output of a comparative sensitivity analysis, where the relative importance of variables considered in the analysis are compared [18].

**Table 1:** Sensitivity analysis inputs.

Parameters	Base-case values	Range for one-way sensitivity analysis
Influenza incidence rate (%)	10.3 [12]	5–14 [13, 14]
Annual Cost of Mild Influenza (RM)	2,435.78	241.93–4,629.63
Annual Cost of Moderate Influenza (RM)	6,504.55	4,764.30–8,244.79
Annual Cost of Severe Influenza (RM)	13,282.66	7,551.90–19,013.42
**Total Annual Cost of Influenza (RM)**	**3,282,833,703.72**	**1,594,618,248–4,464,931,095**

## Results

### Direct cost of influenza

Table 2 lists the characteristics of the influenza patients from the Casemix database. Most of the hospitalised cases were aged <18 years (90.6%) while 77 cases (4.8%) were from among older adults. Similarly, 78.6% (320/407) of outpatient cases were aged <18 years while older patients accounted for 5.2% (21/407) of the sample. Male patients dominated the samples for both service care types. More than half of the inpatient cases had mild influenza (56.5%), followed by moderate (35.1%) and severe cases (8.4%). However, a high number of severe cases (46.7%) was observed among older patients.

**Table 2:** Cost distribution of influenza patients from Casemix database.

Variables	N (%)	Mean (±SD), RM
**Inpatient**	1599 (100)	4,145.60 (2,347.87)
Age distribution		
<18 years	1448 (90.6)	3,979.78 (1,406.85)
18–59 years	74 (4.6)	5,660.34 (7,462.69)
≥ 60 years	77 (4.8)	5,870.38 (4,354.13)
Gender		
Male	940 (58.8)	4,227.66 (2,805.07)
Female	659 (41.2)	4,035.83 (1,461.89)
Severity level distribution		
Mild	903 (56.5)	3,677.08 (671.19)
Moderate	561 (35.1)	4,200.24 (1,607.05)
Severe	135 (8.4)	7,087.95 (6,457.49)
Severity in the older adults (≥ 60 years)		
Mild	13 (16.9)	4,455.15 (1,795.84)
Moderate	28 (36.4)	4,589.82 (1,233.81)
Severe	36 (46.7)	7,377.42 (5,873.24)
**Outpatient**	407 (100)	110.16 (57.15)
Age distribution		
<18 years	320 (78.6)	113.23 (56.78)
18–59 years	66 (16.2)	102.56 (53.70)
≥ 60 years	21 (5.2)	87.24 (67.89)
Gender		
Male	222 (54.5)	116.90 (53.43)
Female	185 (48.5)	102.08 (60.46)

From the Casemix database, the total mean costs were RM4,145.60 for inpatients and RM110.16 for outpatients. The mean cost of influenza hospitalisations increased with age, where it was RM3,979.78, RM5,660.34, and RM5,870.38 for patients aged <18 years, 18–59 years, and ≥60 years, respectively. Contrastingly, the mean cost of outpatient visits was lower for older patients (RM87.24) and those aged 18–59 years (RM102.56) as compared to those aged <18 years (RM113.23). In total, the hospitalisation cost increased as the severity progressed, where it was RM7,087.95 for severe influenza, followed by RM4,200.24 and RM3,677.08 for moderate and mild influenza, respectively. Similarly, the cost was higher for older adults severe cases (RM7,377.42) as compared to the other severity levels, where it was RM4,589.82 and RM4,455.15 in older adults moderate and mild cases, respectively.

The developed clinical pathways for influenza [16] enabled estimation of the direct cost of influenza by the service care type and severity level from the provider’s perspective. Table 3 presents the annual total cost per patient from the calculated cost composition in inpatient and outpatient care. For inpatient care, the direct cost per episode was higher for severe influenza with pneumonia (RM18,179.94) as compared to moderate influenza (RM7,411.31). In moderate cases, two outpatient visits increased the cost to RM833.43 as compared to one visit in mild cases (RM241.93). Overall, the annual cost per patient was significantly higher in severe influenza with pneumonia (RM19,013.42) than in moderate (RM8,244.79) and mild cases (RM241.93). The clinical pathway costing analysis results demonstrated that the overall total costs of influenza were primarily driven by inpatient cost, specifically the ward services cost.

**Table 3:** Cost per patient per year of influenza by type of severity from clinical pathway costing analysis (RM).

	Mild	Moderate	Severe with pneumonia
**Inpatient care cost**			
Administration	-	782.99	1,826.98
Maintenance	-	192.91	450.12
IT Centre	-	107.34	250.47
CSSD	-	15.85	36.97
Dietetic & Food Services	-	261.01	609.03
Medical Record	-	6.44	15.03
Medicine	-	153.66	280.71
Investigation	-	668.36	2,524.21
Physiotherapy	-	238.78	557.16
ICU	-	1,635.47	3,816.09
Ward Services	-	3,348.50	7,813.17
Total direct cost per patient per episode	-	7,411.31	18,179.94
**Outpatient care cost**			
Operational cost	225.00	450.00	450.00
Medicine	16.93	122.02	122.02
Investigation	-	261.46	261.46
Total direct cost per patient per episode	241.93	833.48	833.48
**Total cost per patient per year**	**241.93**	**8,244.79** *	**19,013.42** *

*Total cost per year was included moderate outpatient cost as recommended by the expert

Abbreviation: CSSD; Central Sterilization Services Department, ICU; Intensive care unit

### Economic burden of influenza

Table 4 presents the estimated total annual cost of influenza among older patients in Malaysia. The estimated average annual direct costs of managing mild, moderate, and severe influenza were RM2,435, RM6,504, and RM13,282, respectively. The total economic burden from the clinical pathway costing (RM4.36 billion) was greater than that of the casemix costing (RM2.21 billion). Together, the estimated total annual economic burden of influenza on older patients in Malaysia was RM3.28 billion, which was equivalent to 10.7% of the Ministry of Health Malaysia (MOH) 2020 budget [19].

**Table 4:** Estimated annual cost of influenza among the older population in Malaysia (RM).

Parameter	Casemix cost	Clinical Pathway cost	Average cost
Annual Cost per patient (RM)			
Mild	4,629.63*	241.93	2,435.78
Moderate	4,764.30*	8,244.79	6,504.55
Severe	7,551.90*	19,013.42	13,282.66
Weightage Annual Cost per patient (RM)			
Mild	782.41	40.89	411.65
Moderate	1,734.21	3,001.10	2,367.76
Severe	3,526.73	8,879.26	6,201.50
**Total Annual Cost of Influenza (RM)**	**2,209,053,766.64**	**4,357,629,827.10**	**3,282,833,703.72**
Mild	285,997,957.68	14,946,711.43	150,472,334.56
Moderate	633,913,828.03	1,097,006,008.10	865,498,299.21
Severe	1,289,141,980.93	3,245,677,107.57	2,266,863,069.96
Populations in Malaysia (≥60 years old) [17]	3,548,880		
Influenza Incidence rate (%) [12]	10.3		
Estimated population with Influenza	365,535		

* Annual cost was included two visits of outpatient cost in casemix for ≥60 years.

### Sensitivity analysis

The one-way sensitivity analyses results revealed that the influenza incidence rate exerted the most substantial effect on the total economic burden of influenza, followed by the annual cost of severe influenza. Notably, in the scenario in which the influenza incidence rate was reduced to 5%, the estimated total economic burden was RM1.59 billion. Meanwhile, if the influenza incidence rate increased to 14%, the total economic burden of influenza was projected to be RM4.46 billion. By contrast, the annual cost of mild influenza exerted the fewest effects on the total economic burden, where it ranged from RM3.15 billion to RM3.42 billion (Fig 1).

## Discussion

We analysed influenza treatment costs with casemix costing and clinical pathway costing. Both methods covered inpatient and outpatient service care and influenza severity. In clinical pathway costing, outpatient care was separated into mild and moderate cases, whereas casemix costing did not capture outpatient care based on the severity of illness. The designed influenza clinical pathways for inpatients were divided into moderate and severe with pneumonia in older adults. Conversely, casemix data contain all degrees of severity (mild, moderate, severe) of influenza for inpatient cases. The casemix costing uses a top–down costing approach that involves only a few data sources and time, which yield a limited level of detail [18] compared to the activity-based costing approach that requires extensive data sources. A combination of both costing methods is recommended as a pragmatic approach, especially in the absence of systematic cost data collection.

In this study, census samples were obtained from the casemix data from a teaching hospital in Malaysia for cases in 2010–2020 for inpatient, whereas outpatient case data is limited to the years 2019 and 2020, representing a two-year collection period. It is important to note that the hospital’s only available outpatient dataset covers this two-year span, while the inpatient data spans a full ten years. Most of the influenza patients were young and had mild influenza. A study in England reported that the highest influenza-attributable hospital admission rates were among healthy children aged <5 years, and nearly 40% of the hospital admissions and consultations for influenza involved children aged <15 years [20]. By contrast, the older patients in the present study had higher rates of severe influenza. Older people, particularly those with chronic illnesses and/or frailty, are susceptible to severe influenza outcomes. Additionally, a recent report suggested that the influenza mortality rate was highest among adults aged >70 years, especially those with underlying comorbidities [7].

On average, the outpatient cost in the casemix data of older patients was RM87.24 (USD20.77), which was lower than the RM241.93 (USD57.60) cost per visit for outpatient services in the clinical pathway. The differences in the two costs were simply due to differences in costing methodology and reflected different levels of accuracy. However, these values were comparable with that of a systematic review, where most of the studies reported a cost between USD1.09 and USD39.31 for outpatient services cost per influenza-like illness (ILI) episode [21]. The cost of inpatient influenza treatment was presented according to the degree of severity. This study demonstrated that the inpatient cost per episode for influenza management was significantly higher in severe cases for both the casemix and clinical pathway, where the cost was RM7,087.95 (USD1,687) and RM18,179.94 (USD4,329), respectively. Patients with severe influenza require further investigation and intensive treatment to reduce their morbidity and mortality risk. Therefore, this would increase the hospitalisation LOS due to the influenza-related complications. Overall, the mean cost of influenza treatment in casemix costing for older patients was RM5,870 (USD1,397), which was slightly lower compared to the average cost in other countries, where wide variations between hospitalisation costs persist and range from USD1,549 in Hong Kong to >USD7,000 in most US studies [21]. Inpatient admissions consume many healthcare system resources. Consequently, various hospitalisation rates and the unit cost associated with each hospitalisation event could be potentially relevant sources of variation. In the casemix costing, higher cost was also observed in older patients as compared to those from other age groups. A previous study reported a high percentage of at-risk patients among older patients with multiple chronic diseases [22]. The presence of underlying comorbid conditions and the declining immune competence that accompanies ageing further increase the influenza infection risk among older adults. The risk of hospitalisations was also increased with the existence of comorbidities [23, 24]. A study reported that the direct medical cost of hospitalised influenza was higher for those aged 65 and older with comorbid diseases such as chronic cardiac, pulmonary, renal, diabetes mellitus, and others compared to those at low risk [25].

In the present study, the influenza annual cost was estimated by incorporating both costing approaches, where two outpatient visits were assumed prior to hospital admission in accordance with the published clinical pathway for outpatient moderate cases [16]. This study highlights the fact that the average annual costs per patient for mild, moderate, and severe influenza were RM2,435.78 (USD580), RM6,504.55 (USD1,549), and RM13,282.65 (USD3,163), respectively. Mild cases incurred lower costs, given that there were no admissions in the clinical pathway costing. Consequently, this resulted in a wide range of costs between illness severity and service care type. Furthermore, the LOS greatly affected the influenza cost, i.e. 14-day hospitalisation for severe influenza with pneumonia as compared to moderate cases (7 days) due to the comorbidities and influenza-related complications [16]. Our results were slightly lower than those of a previous study that reported mean estimated costs of USD8,330 for hospitalised patients aged 65–84 years [11]. Meanwhile, a recent study reported comparable results, with the average cost of hospitalisation per person being USD3,274 in the influenza-positive group, which included adults aged ≥18 years in the study population [26]. Our results indicated that influenza imposed a substantial annual economic burden. In Malaysia, the total annual economic burden of influenza among older patients was between RM2.21 billion (USD526 million) and RM4.36 billion (USD1.04 billion) for casemix and clinical pathway costings, respectively. There were significant differences in the total annual economic burden between the two costing approaches. The clinical pathway costing was conducted using mixed methods, which are feasible, rapid, and accurate as compared to the full top–down costing approach. A previous study reported a 21–36% lower median cost in full top–down costing and carried the risk of underestimating assessed costs in comparison to the mixed method [27]. Given that bottom–up costing yields more detailed information on the disease cost and consequently on the utilisation of health-allocated resources, it is believed that the combined use of these two costing techniques compensates for limitations in either technique. Therefore, the average annual cost was calculated to estimate the average total economic burden to provide reliable and comprehensive information for stakeholders and policymakers in Malaysia.

Overall, we demonstrated that the average total economic burden of influenza was RM3.28 billion (USD782 million), which is 10.7% of the total MOH budget in 2020 [19]. This estimate suggested that the substantial costs from influenza would affect the total Malaysian budget and gross domestic product (GDP). However, our result was lower than estimates in the US, which ranged from USD6.3 billion to USD25.3 billion [11]. In the present study, we focused on the direct medical costs of influenza-related hospitalisations in older patients. Therefore, there were various differences between previous studies from other countries as compared to our results [25, 28]. Echoing our findings, a Chinese study that focused on older patients also highlighted a lower economic burden as compared to the estimated burden in the US [29]. Furthermore, those studies reported varied estimations of the number of events attributable to influenza, which would influence the unit costs linked to these events. The burden of influenza is also associated with the size of the older population and the influenza attack rate. Given that populations are ageing worldwide, the burden of influenza on older adults are likely to increase in the coming years if prevention strategies are not implemented. Future studies should include direct non-medical costs and indirect costs, including transportation, to accurately measure the annual social and economic burdens and effects attributable to influenza infections in Malaysia. Preventive measures such as immunization against influenza, especially for older adults, can aid reduction of the economic burden of this disease in the country.

The sensitivity analysis results indicated that the total economic burden of influenza was most sensitive to the influenza incidence rate. In the estimation, we used the baseline rate from a recently published influenza-related disease burden in Malaysian adults [12]. This highlighted the uncertainty in influenza disease burden estimates, especially among older people. In Malaysia, influenza is not a notifiable disease and limited data on cases have been collected through the surveillance system. Due to the variability of data collection and diagnostic testing methods used, the disease burdens reported in East and Southeast Asia vary widely [14]. Meanwhile, the cost of influenza exerted the fewest effects on the total economic burden of influenza at all severity levels. In this study, the annual cost on older adults was estimated using a weightage cost based on the number of patients by severity level in casemix data. The estimation was conducted assuming the influenza incidence rate included all influenza severity levels.

This study has a few limitations. First, the cost of productivity losses was not included, which might have influenced the outcome. It was decided to concentrate on the direct medical costs due to the data limitations at the time of study design. Previous study reported higher overall direct costs per case in the older than in younger adults [30]. Indirect costs were insignificant and could be related to the lower employment rates in older adults. However, a recent review in Thailand reported that 50–53% of economic burden estimates referred to lost productivity [31]. Societal perspectives enable a comprehensive analysis of all opportunity costs associated with a disease. We encourage researchers to further expand upon the findings given in this study, as more data becomes accessible in order to provide a comprehensive understanding of the healthcare implications connected with influenza. Nonetheless, the use of the provider’s perspective in the present study was appropriate as we intended to quantify the medical cost of the diseases. Apart from that, the total economic burden was estimated based on the estimated influenza incidence rate for all age groups. Future research should be conducted to estimate the influenza incidence rate only for targeted populations in Malaysia, such as older people or children. However, the sensitivity analysis was conducted to overcome these shortcomings and to investigate the parameter variability.

In this study, the cost data was collected from a teaching hospital in Malaysia which might not be generalised to other public and private hospitals in Malaysia. Cost of medical procedures and hospitalisation are often depending on the hospital type, management preference and outcome of procedures [32]. To our knowledge, this is the first study in Malaysia presenting the economic burden of influenza in older adults. Despite its limitations, we believe that this study provides a better understanding of the economic burden of influenza and establishes baseline information for more complex economic evaluation in the future. Characterizing the influenza-related economic burden is fundamental to cost-effectiveness analyses, which are essential for the implementation of influenza vaccination programmes by policymakers. Although a comprehensive cost-effectiveness analysis of influenza vaccine is currently lacking in Malaysia, studies and reviews from other countries consistently demonstrated that it is cost-effective. The provision of free influenza vaccination to the high-risk and older population in Malaysia may have been influenced by various factors, including healthcare policies, budget constraints, and public health priorities. However, advocacy campaigns, initiatives and evidence-based are always the keys to support the implementation of influenza vaccination programmes [33].

## Conclusions

This study highlighted the significant economic burden imposed by influenza on the healthcare system. The estimation of the economic burden will provide crucial information to decision-makers regarding the implementation of immunisation programmes to reduce the burden and complications of influenza among older people in Malaysia. The findings also formed the foundation for economic evaluation analysis to identify the most efficient and effective methods for minimising influenza in older adults and its contribution to the annual economic burden of influenza.
